# Haloarchaea as Cell Factories to Produce Bioplastics

**DOI:** 10.3390/md19030159

**Published:** 2021-03-18

**Authors:** Lorena Simó-Cabrera, Salvador García-Chumillas, Nashwa Hagagy, Amna Saddiq, Hend Tag, Samy Selim, Hamada AbdElgawad, Alejandro Arribas Agüero, Fuensanta Monzó Sánchez, Verónica Cánovas, Carmen Pire, Rosa María Martínez-Espinosa

**Affiliations:** 1 Biochemistry and Molecular Biology Division, Department of Agrochemistry and Biochemistry, Faculty of Science, University of Alicante, Carretera San Vicente del Raspeig s/n-03690 San Vicente del Raspeig, 03690 Alicante, Spain; lorena.simo@ua.es (L.S.-C.); carmen.pire@ua.es (C.P.); 2 Centro Tecnológico del Calzado y del Plástico de la Región de Murcia, Av. Europa 4-5, 30840 Alhama de Murcia, Spain; schumillas@gmail.com (S.G.-C.); a.arribas@ctcalzado.org (A.A.A.); f.monzo@ctcalzado.org (F.M.S.); v.canovas@ctcalzado.org (V.C.); 3 Department of Biology, College of Science and Arts at Khulis, University of Jeddah, Jeddah 21921, Saudi Arabia; niibrahem@uj.edu.sa (N.H.); hmtaha@uj.edu.sa (H.T.); 4 Botany and Microbiology Department, Faculty of Science, Suez Canal University, Ismailia 41522, Egypt; 5 Department of Biology, College of Science, University of Jeddah, Jeddah 21921, Saudi Arabia; aansaddiq@uj.edu.sa; 6 Zoology Department, Faculty of Science, Suez Canal University, Ismailia 41522, Egypt; 7 Department of Clinical Laboratory Sciences, College of Applied Medical Sciences, Jouf University, Sakaka, P.O. 2014, Saudi Arabia; sabdulsalam@ju.edu.sa; 8 Botany and Microbiology Department, Faculty of Science, Beni-Suef University, Beni-Suef 62521, Egypt; Hamada.abdelgawad@science.bsu.edu.eg; 9 Multidisciplinary Institute for Environmental Studies “Ramón Margalef”, University of Alicante, Ap. 99, E-03080 Alicante, Spain

**Keywords:** bioplastics, polyhydroxyalkanoates (PHA), polyhydroxybutyrate (PHB), polyhydroxyvalerate (PHV), haloarchaea

## Abstract

Plastic pollution is a worldwide concern causing the death of animals (mainly aquatic fauna) and environmental deterioration. Plastic recycling is, in most cases, difficult or even impossible. For this reason, new research lines are emerging to identify highly biodegradable bioplastics or plastic formulations that are more environmentally friendly than current ones. In this context, microbes, capable of synthesizing bioplastics, were revealed to be good models to design strategies in which microorganisms can be used as cell factories. Recently, special interest has been paid to haloarchaea due to the capability of some species to produce significant concentrations of polyhydroxyalkanoate (PHA), polyhydroxybutyrate (PHB), and polyhydroxyvalerate (PHV) when growing under a specific nutritional status. The growth of those microorganisms at the pilot or industrial scale offers several advantages compared to that of other microbes that are bioplastic producers. This review summarizes the state of the art of bioplastic production and the most recent findings regarding the production of bioplastics by halophilic microorganisms with special emphasis on haloarchaea. Some protocols to produce/analyze bioplastics are highlighted here to shed light on the potential use of haloarchaea at the industrial scale to produce valuable products, thus minimizing environmental pollution by plastics made from petroleum.

## 1. Introduction

Plastic pollution is distributed across the globe, and it is a problem of growing environmental concern, which is aggravated by the great durability of polymers and their inaccurate management [1]. Global production of resins and fibers increased from 2 metric tons (Mt) in 1950 to 380 Mt in 2015. From that date, approximately 6300 Mt of plastic waste has been generated, around 9% of which was recycled, 12% was incinerated, and 79% ended up in landfills or the natural environment, with its consequent accumulation [2]. More than 80% of the plastics produced are obtained through polymerization of monomers into high-molecular-weight chains, receiving the name of thermoplastics [3,4]. The physical (e.g., melting, extrusion, and pelletization) and chemical (mixture with antioxidants, plasticizers, clarifiers, bisphenol A-based polycarbonate, copolymers, colorants, etc.) properties of these polymer matrixes are modified, thus obtaining a complex chemical composition in their physical structure [4]. In general, these materials show high malleability which, together with their low cost and versatility, have contributed to an increase in their use worldwide. It is estimated that plastics now flow through major food-webs across the Earth, with potential implications for populations and ecosystems as well as human health [5]. Plastic waste has affected over 690 marine species with small plastic particles ending up in the digestive tract of organisms from different trophic levels [6]. Under different conditions, such as mechanic abrasion, temperature fluctuations, or the influence of light or sea waves, plastics go through a fragmentation process into smaller particles named microplastics, whose size is below 5 mm [7].

Microplastics are ubiquitous in water, soil, and atmospheric environments and come from different sources. The discharge of microplastic-containing wastewater is a major source of microplastic pollution in water [8]. Carr and co-workers stated that although sewage treatment systems present a removal rate of microplastics as high as 98%, 65 million microplastics still enter water through these facilities every day [9]. The abundance of microplastics ranged from 741 to 7707 items·kg^−1^, with the minimum value corresponding to 1971 and the maximum value to 2018. The outer layer of microplastics is ideal for harboring bacteria, viruses, and algae, and abiotic substances [10]. These abiotic substances include organic pollutants, perfluorinated compounds (PFCs), heavy metals, and pharmaceuticals and personal care products (PPCPs). Consequently, the adhesion of pollutants makes microplastics a source of pollution [8]. The long-distance microplastic migration also increases the risk of biological invasion, as attached microorganisms follow the same route [11]. A major problem about microplastics is their capacity to release absorbed contaminants and chemical additives into an organism, compromising its health [12]. Due to their small size, microplastics are ingested by filter, suspension, and detritus feeders living in the water column and bottom sediments, and they are present in the guts of invertebrates, fish, turtles, and a range of species intended for human consumption or involved in critical ecological roles [13]. Microplastics can potentially harm animal species, since many plastic additives and persistent chemicals are endocrine disruptors, altering metabolic and reproductive endpoints [14]. These substances are known as endocrine disrupting chemicals (EDCs), which affect all hormonal systems controlling the development and function of reproductive organs, regulation of metabolism, and satiety [15]. Bisphenol A (BPA) and bis(2-ethylhexyl)phthalate (DEHP) are EDCs used as plastic components or additives, which can cause chronic adverse effects on several organisms [16]. It is also known that floating microplastic fibers and their adsorbed pollutants enter the human body via the respiratory system, causing a lasting physiological impact on the body [8].

Due to the considerable negative impact of the production, use, and disposal of plastics, not only the scientific community but also political sectors are stressing the need to look for alternatives that allow the use of biodegradable plastics and their production following environmentally friendly procedures. Unlike fossil-fuel plastics, which are derived from petroleum, bioplastics are a form of plastics derived from renewable biomass sources. Biodegradability is a property that enables certain bioplastics, but not all, to be chemically broken down by the action of microorganisms, such as bacteria, fungi, and algae. Therefore, bioplastics are an alternative to synthetic plastics to reduce their environmental impact. They are produced by several microorganisms when there is availability of excess substrates (carbon source) under conditions of limited oxygen, nitrogen, phosphorus, or pH fluctuations [17]. As an example, polyhydroxyalkanoates (PHAs) are biological polyesters mainly produced by microbial fermentation processes. They are recognized because of their 100% biodegradability, biocompatibility, and sustainability [18]. The first identified PHA was polyhydroxybutyrate (PHB), which is utilized as a reserve material in bacteria, amounting up to 80% of the dry bacterial biomass [19,20].

The interest in identifying the molecular machinery sustaining the microbial production of bioplastics is such that the number of scientific publications on this topic has increased significantly over the last decade (more than 9000 research articles and reviews focused on plastics pollution and potential solutions have been published over the last 10 years) [21].

In this context, microbes able to synthesize bioplastics are considered good models to develop biotechnological-based processes in which microorganisms can be used as cell factories for bioplastic production at a large scale. Extremophilic microbes in general, and particularly the haloarchaea group (Archaea domain), have received considerable attention from the scientific community due to their peculiar metabolic capabilities, including the capacity of bioplastic production shown by some species. Thus, some haloarchaea can produce significant concentrations of marketed bioplastics, such as polyhydroxyalkanoate (PHA), polyhydroxybutyrate (PHB), and polyhydroxyvalerate (PHV). The growth of those microorganisms at the pilot or industrial scale offers several advantages compared to other microbes that are bioplastic producers in terms of sterilization of the cultures, growth rate, etc. This review summarizes the most recent findings regarding the production of bioplastics by microorganisms, with special emphasis on haloarchaea as promising organisms, to reduce the negative effect of plastics contamination in the future. Discussions about protocols already used to produce and analyze bioplastics are also highlighted to shed light on the potential use of haloarchaea at the industrial scale in order to produce those valuable products.

## 2. Description of Bioplastics; PHA, PHB, PHV, and PHVB as Examples

Bioplastics are plastics produced by microorganisms, most of them showing high biodegradability (they could be chemically broken down by the action of microbes). Most biodegradable bioplastics are aliphatic polyesters composed of certain types of monomers and isomers. The different variables of the polymerization process will determine the distribution of the monomers, length, and branching of the polymer chain. Like other thermoplastics polymers, biodegradable bioplastics are semicrystalline polymers characterized by their molecular weight, distribution of branches, melting temperature (T_m_), and/or glass transition temperature (T_g_). Physicochemical properties of biodegradable bioplastics do not only depend on the chemical composition and structure of the polymer chain. The processing temperature and annealing time have a direct effect on the degree of crystallinity, which ultimately impacts the general properties of biodegradable bioplastics. For instance, mechanical, optical, barrier properties; rate of biodegradation; thermal–chemical resistance; and resistance to aging are influenced to a greater or lesser extent by the mentioned processes [22]. They are water insoluble, and their sinking properties in water facilitate biodegradation in the absence of oxygen in sedimentary soil [23]. All these physicochemical properties are discussed into detail in the following subsections.

### 2.1. Chemical Structures

Among bioplastics, PHAs are biocompatible and biodegradable polyesters produced by microorganisms, whose biodegradability depends on the nature and chemical composition of their constituents as well as on the environmental conditions and the microbial mechanisms of biodegradation. These polymers have been attracting attention over the last decades as they are an environmentally friendly alternative to plastics manufactured from petrochemicals [24,25].

**Figure 1:**
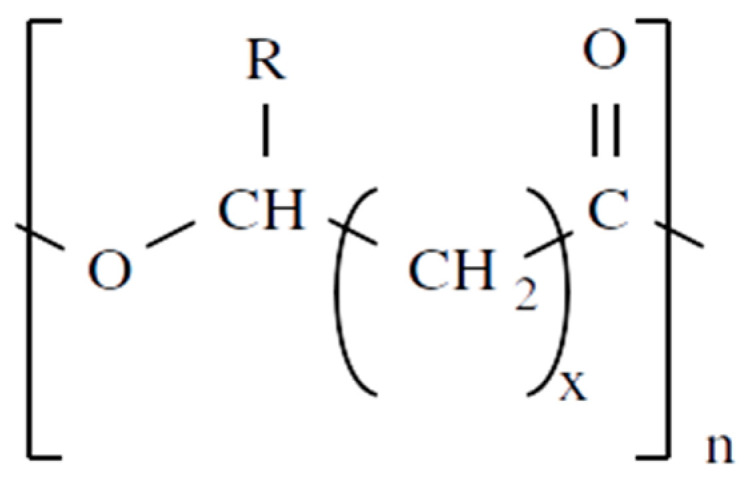
Chemical structure of polyhydroxyalkanoates (PHAs).

Unlike other biodegradable bioplastics, the entire biosynthesis process of PHAs, both the production of monomers and polymerization, occurs within microbial cells (they are usually formed under instable growth conditions as intracellular carbon and energy reserves by extensive assortment of microorganisms) [26]. PHA molecules have been predominantly recognized as aliphatic/linear complexes that are optically active biopolyoxoesters and composed of monomers known as (R)-3-hydroxy fatty acid monomer units, with a side chain R group (Figure 1) [25]. The latest is usually a saturated alkyl group, although it can also undertake fewer common forms, such as unsaturated alkyl groups, branched alkyl groups, or substituted alkyl groups [27].

Physical and chemical properties of PHA polymers differ from each other depending on the chemical and structural composition of their monomeric unit [17] (Table 1). Thus, these polymers can be classified into different groups based on different criteria of classification. If the criterion for classification is focused on the chemical nature of the monomers, PHAs are classified as following [28,29]:

1. PHA containing aliphatic fatty acids, such as PHB.
2. PHAs containing aromatic fatty acids, such as poly(3-hydroxy-5-phenylvalerate) PHPV.
3. PHA heteropolymers containing both aliphatic and aromatic fatty acids (i.e., P(3HB-co-3MP)).

Based on the number of carbon atoms in the monomer units, PHAs can be classified into three classes: short-chain length PHAs ((scl)-PHAs; C3 to C5), for example P3HB or P4HB; medium-chain length PHAs ((mcl)-PHAs; C6 and C14), such as P3HHx and P3HO; and long-chain length PHAs ((lcl)-PHAs; more than C14) [30,31]. Depending on the chemical composition of the monomers and their distribution in the polymer chain, PHAs can be classified as follows [30]:

1. Homopolymers: composed of a single type of monomer, such as poly 4-hydroxybutyrate (P4HB), poly 3-hydroxypropionate (P3HP), poly 3-hydroxyvalerate (PHV), poly 3-hydroxy-4-pentenoate (P3H4P), poly 3-hydroxyhexanoate(P3HH), or poly 3-hydroxyheptanoate (P3HH).
2. Random copolymers: composed of more than one type of randomly distributed monomers. The wide range of commercially produced PHA as random copolymers include poly(3HP-co-4HB), poly(3HB-co-3HP), poly(3HB-co-3HV) (PHBV), and poly(3HB-co-4HB) (P3HB4HB).
3. Block copolymers: these molecules are characterized by chemically distinct monomer units grouped in discrete blocks along the polymer chain. Different di-block copolymers have been produced by means of regulating the availability of fed substrates, such as PHB-b-P3HVHHp, PHB-b-P4HB, PHB-b-PHHx, P3HB-b-P3HP, P3HP-b-P4HB, and P3HHx-b-P(3HD-co-3HDD).

PHAs are water-insoluble, and their sinking properties in water facilitate biodegradation in the absence of oxygen in sedimentary soil [23]. Solubility of these polymers is higher in chlorinated solvents and null in the non-chlorinated type [17]. PHB, the first PHA characterized, shows a behavior like that of polypropylene, being useful for gas entrapment and moisture resistance [32]. Its homopolymer has a high degree of crystallinity, which gives it a stiff and brittle nature. It can involve environmental and sustainable advantages despite its blending with petrochemical-based polymeric matrices. showing low degradation values due to its mechanical characteristics. The amount of PHB in the blends adjusts the microstructure and can vary its characteristics, such as hydrophobicity, crystallinity, or flexibility [33]. The main chemical properties of PHAs are displayed in Table 2.

### 2.2. Physical Properties

The effect of chemical composition on the basic properties of PHAs polymers is still under research. Discrepancies in results obtained from different studies are prone to controversies and are matter of debate. Thermal properties of thermoplastics polymers are defined by the glass-transition temperature T_g_ and the melting temperature T_m_. Like most thermoplastic polymers, PHAs are semi-crystalline polymers with a crystal structure formed by multi-oriented lamellar crystals surrounded by disordered polymer chains forming the amorphous phase. The T_g_ is a property of the amorphous region, the temperature at which the amorphous phase turns from a glassy state to a rubbery state, determining the mechanical properties of the polymer. PHAs with T_g_ values over room temperature are brittle, stiff, and rigid, due to lack of mobility of their polymer chains. The T_m_ is a property of the crystalline phase, and it is the temperature at which this phase turns from a solid state to a liquid state; it defines the behavior of the molten polymer during processing. Furthermore, the degree of crystallinity of the polymer is also closely related to its mechanical strength. It can be concluded that the conditions of the melting process depend on one hand, on the melting temperature (T_m_) and thermal degradation temperature (T_degr_) of the polymer; on the other hand, the degree of crystallinity and glass transition temperature (T_g_) determine the following final properties of the polymer:

1. Biodegradability: unlike any other types of biodegradable plastics, PHAs can biodegrade under anaerobic conditions. In all cases, PHAs’ degradation rate can be accelerated by means of lowering crystallinity [36] and melting temperature [37]. PHA copolymers containing the 4HB monomer seem to degrade more rapidly than P(3HB) or P(3HB-co-3HV) copolymers [38].
2. Mechanical properties: Young’s modulus, tensile strain and stress, and elastic modulus provide a measure of PHA’s stiffness. The latest varies from that of very stiff polymers, such as PLA and PP, and from much softer material, such as LDPE. The incorporation of 3HA units into the PHB polymer chain tends to decrease the stiffness and simultaneously increase the ultimate elongation of the material. These effects are more pronounced with mcl-3HA having longer side chains. Lower crystallinity and T_g_ lead to softer materials. The decrease in the size of the crystals in the crystalline phase also causes an improvement in the elongation to break and impact strength [39].
3. Gas barrier properties: the lower the crystallinity, the lower the gas permeability of the material. Despite this, the gas barrier properties of PHAs are insufficient to explore potential applications at a large scale. Consequently, research is underway to improve this feature for packaging applications [40,41].
4. Transparency: optical transparency is enhanced when the amorphous portion in the polymer is increased [34]. Few articles have been found that refer to this property in PHAs [36,42].

Another important property to be analyzed is hydrophobicity, which affects solubility, biodegradability, and biocompatibility. PHAs are slightly hydrophilic materials, soluble in chlorinated solvents such as chloroform or 1,2,2-tetrachloroethane and insoluble in water, ethanol, or methanol. Solubility is an important property to consider when selecting the suitable solvent for the preparation and purification of the polymer [42]. Biodegradation of PHAs is influenced by the hydrophilicity–hydrophobicity balance and accessibility to their surface by PHA-depolymerizing enzymes [36]. Hydrophobicity can be modified by graft polymerization introducing polar groups into the polymer chain [43,44].

The most studied PHA is P3HB, characterized by its intrinsic brittleness due to a degree of crystallinity higher than 70% [45]. This distinctive property is attributed to two processes: the re-crystallization of the amorphous phase during storage at room temperature and the glass transition temperature (T_g_) being close to room temperature. Thus, mechanical properties change with time, becoming, after storing at room temperature, more rigid with a decrease in the values of elongation at break for 30 days [34]. PHBs are linear polymers lacking branches. The absence of ramifications contributes to their poor melt elasticity, low thermal degradation temperature, and high crystallinity.

It has been reported that an increase in the length and variety of the side chains hinder the formation of the crystalline phase and reduce the value of T_g_ and degree of crystallinity of PHAs [37]; mechanical properties of PHAs can be improved by long chain branching (lcb) and polymer chain entanglement. (scl)-PHAs exhibit properties that depend on their monomeric composition. For instance, P3HB (with a T_m_ around 180 °C and a T_g_ of 4 °C) exhibits high crystallinity, brittleness, and stiffness, while P4HB, which possesses a T_m_ of 54 °C and a T_g_ of −49 °C, is a malleable thermoplastic material whose tensile strength is comparable to that of polyethylene. Differently, mcl-PHAs are elastomers, whose T_m_ values are between 40 °C and 60 °C and T_g_ values between −50 °C and −25 °C, and they enhance their low crystallinity, low tensile strength, and high elongation to break [30,37,46,47].

To predict PHAs properties, it is essential to analyze their molecular weight, which is related to the length of the polymer chains, and this will determine both the behavior of the PHA during use and in processing. An increase in molecular weight results in an increase in melt viscosity and an improvement in mechanical properties [47,48].

Finally, biocompatibility of the biopolymers is a property required for medical applications. Regarding PHAs, P(3HB-co-3HV) polymers have shown good biocompatibility both in vitro and in vivo [37]. Block copolymerization of PHAs with polyethylene glycol (PEG) has been shown to be a promising way to decrease hydrophobicity and adjust biodegradation rates for biocompatibility requirements [44].

### 2.3. Improving PHAs’ Physicochemical Properties by Copolymerization to Promote Industrial Uses

One of the main approaches to improve the properties of PHAs is the production of derivatives based on the biosynthesis of copolyesters. Thus, due to the poor physical properties of the PHB polymer, exertions are focused on the synthesis of co-polymers that have more enhanced physical properties than PHB does. Results recently reported have demonstrated that incorporation of hydroxyvalerate (HV) into PHB polymers results in a poly(3-hydroxybutyrate-co-3-hydroxyvalerate) (P(3HB/3HV)), which is less stiff and brittle than pure P(3HB) as well as possessing a lower melting point [49,50]. The temperature characteristics and degrees of crystallinity of PHAs are affected by the chemical compositions of the monomers and their quantitative contents in the polymers. For instance, the incorporation of 4HB, 3HV, and 3HHx units into the chain of poly(3HB) decreases its melting temperature [51]. Hence, the higher the content of the second monomer units, the greater the changes [45]. The incorporation of 3HV or 3HHx monomers into the 3HB chain also decreases to some extent the degree of crystallinity as previously mentioned, while the incorporation of 4HB into the 3HB polymer chain has been found to have a dramatic effect (much more significant than that of the incorporation of 3HV or 3HHx) on the ratio of the crystalline and amorphous phases in the copolymer [51]. These comonomers improve the plastic flexibility by lowering the glass transition temperature (T_g_) and the melting temperature (T_m_) [46,52]. Copolymers with up to four different monomers have been studied to determine the effect of the composition and ratio of monomers on the final properties of the polymer [51,53,54]. However, studies on the effects of the different monomers and their concentrations on the final properties of the polymer are still scarce.

A class of PHA random copolymers called NodaxTM [54] include (mcl)-3HA units (3HHx, 3HO, 3HD) in the 3HB polymer backbone, which leads to a moderately branched polymer. The branches act as a molecular defect, which disrupts the excessive regularity of the polymer chain and consequently lowers the melt temperature (T_m_) and crystallinity. The incorporation of (mcl)-3HA comonomer units in the PHB polymer chain also reduces the T_g_ of copolymers, being more effectively lowered by the incorporation of 3HA units with longer side chains. As a result, these PHAs are more flexible and ductile.

Regarding the molecular weight of PHA polymers, it has been found that weight-averaged molar mass M_w_ and polydispersity vary in a wide range. Additionally, no correlation has been established with the composition of copolymer polyhydroxyalkanoates [51,53]. PHA molecular weight is a highly variable parameter, which is determined by several factors, such as the type of the PHA-producing microorganism, carbon source, duration of cultivation, and technique of polymer extraction. The types and concentration levels of carbon sources seem to play a major role in determining the molecular weight [55].

## 3. Analysis Used for PHA Isolation and Characterization

### 3.1. Methods for Staining Cells to Distinguish PHA/PHB Producers and Non-Producers

When new microbial isolates are firstly described, easy approaches based on cellular staining can be used to determine whether the cells are able to produce PHAs. Most extended protocols include the use of lipophilic dyes, such as Sudan black B [56], Nile blue A, and Nile red [57]. Some of the main characteristics of these methods are described below.

Sudan black B (C29H24N6) is a diazo fat-soluble dye [58]. PHAs are observed as black granules with Sudan black B by bright-field microscopy [59]. It is carcinogenic and has a toxic effect on humans [60], but it is still widely used in biological and histological works as well as for commercial benefits [61].

Nile blue A and Nile red are highly fluorescent and photostable organic dyes belonging to the benzophenoxazine family, and, historically, due to their intense colors and their lipophilic nature, they have been widely used for staining lipids in vitro [62]. Nile red is a neutral molecule that is poorly soluble in water, and its chromophore is highly susceptible to changes in solvent polarity, showing little or no fluorescence in most popular solvents. Nile blue A, also known as Nile blue sulphate or Basic blue 12, is a cationic dye and is thus more soluble in water than Nile red, presenting a redshifted absorbance spectrum. Both fluorescent dyes are particularly useful for visualizing hydrophobic cell structures, such as membranes or lipid-like inclusions (PHA granules) [63]. PHA inclusions appear as brightly fluorescent red/orange granules with Nile blue A [64] and with Nile red [65].

Red Nile and Nile Blue A have a greater affinity for PHB than Sudan Black B [63,66] and by fluorescence microscopy provides high-quality images. However, weak fluorescence may occasionally occur because of the unspecific binding to lipid membranes or haloarchaeal cell envelopes due to the lipophilic nature of Nile Blue A [66]. For this reason, complementation of fluorescence-based staining with Sudan Black B staining can be suitable for the identification of PHA-producing organisms.

Nile Red, Nile Blue A, and Sudan Black B have been widely used for the screening and identification of PHA inclusions in bacteria [59,67]. More recently, new methods based on Nile red fluorescence staining have been also developed for the quantification of intracellular bacterial PHAs [68]. Although these methods are widely spread for the screening of PHA-producing environmental bacteria, they have been rarely applied for screening of PHA-producing archaea. In 2010, Legat and colleagues used Sudan Black-, Nile blue A-, and Nile red-based techniques for the screening of PHA-producing haloarchaea, demonstrating the utility of these method also in the Archaea domain [66]. They isolated more than 20 PHA-producing haloarchaeal strains amongst the genus *Halococcus* sp., *Haloarcula* sp., *Halobacterium* sp., *Halorubrum* sp., *Haloferax* sp., and *Natronococcus* sp. Of notice, six different species from the genus *Halococcus* were identified for the first time as PHA-producing species. Most of the isolated microorganisms accumulated PHBV copolyesters without the presence of related precursors in the culture medium.

### 3.2. Protocols for PHA Solation: Advantages and Disadvantages

PHA isolation requires various techniques, which can generally be summarized as biomass harvesting, pretreatment, PHA recovery, PHA accumulation, polishing, and drying [52]. As PHAs are intracellular polymers, biomass harvesting involves concentrating biomass via filtration or centrifugation. Prior to the polymer extraction process, biomass must be subjected to a drying step to facilitate PHA recovery. This can be achieved via lyophilization or by thermal treatment, but lyophilization, despite being effective, is difficult to be used on an industrial process because of its economic requirements and its complexity. On the other hand, thermal drying is more affordable and easier to scale industrially [69]. Different processes, such as grinding and chemical and biochemical pretreatments can be combined to increase the finally obtained product purity [70]. PHA recovery can be carried out by different methods: solvent extraction, chemical disruption, enzymatic cell disruption, or mechanical disruption.

Solvent extraction is the oldest method of PHA recovery [20] and consists of the application of solvents to extract the polymer from cells. Its advantages are high product purity and polymer integrity (they are not degraded during the isolation process). Among the disadvantages, it is worth mentioning that it is not economically feasible, it can be hazardous to humans and the environment, and it is not suitable for the industrial scale. Chemical disruption of cells to obtain granules is focused on the use of some chemicals, such as surfactants and sodium hypochlorite, to solubilize membranes and other envelopes, thus releasing intracellular content (including PHAs granules). The use of surfactants shows advantages, such as direct PHA recovery from the cultures and a limited degradation of the polymer, but it has high costs, and SDS is difficult to recover and to remove from the polymer at the end of the process. Sodium hypochlorite also offers a high product purity, and it is applicable to large-scale operations, but it may digest the polymer, losing molecular weight.

Enzymatic cell disruption is another approach, and it involves the use of enzymes, such as proteases, nucleases, lysozyme, and lipases, to cause cell lysis without affecting the polymer [71]. The advantages of this method include low energy requirements and a high recovery rate and purity of the polymer, but the enzymes’ production is of high cost, which is the principal disadvantage for industrial implementation [72].

Mechanical disruption is a widely applied method for disintegration of microbial cells, releasing proteins to the outside. It can also be used for the release of PHA in the laboratory. One of these methods is called high-pressure homogenization (HPH), a non-solvent method for disruption and homogenization of microbial cells [73]. It is advantageous on a large scale, but at low biomass concentrations, cell disruption is low. Another method is the disruption of cells by shear forces caused by solid beads, giving it the name of disruption by bead mill. The advantage is that chemicals are not required, but scaling up the recovery process is difficult, because it requires several steps and efficient cooling to release the heat formed during the mill process. A third method based on mechanical disruption consists of disruption by ultrasonication, which allows one to obtain high purity of the product when combined with other extraction methods; it is not easily applicable at the large industrial scale [71].

There are other methods for PHA recovery in which chemicals are not necessary but genetically engineered production strains are required, which is a disadvantage. Furthermore, other methods such as air classification or dissolved-air flotation allow for high purity of the product and do not require chemicals, but they must be combined with other methods, such as mechanical disruption or enzymatic disruption, increasing the number of steps necessary for polymer recovery [71].

Because PHAs accumulate intracellularly, downstream processing is a particularly important step in the purification and separation of PHA in terms of yield and cost. In fact, a major factor of the costs of PHA production is the recovery of the product from the cellular matter. Generally, the efforts needed to break the cell walls strongly depend on the type of production strain. In this sense, halophilic PHA-producing microorganisms such as haloarchaea offer advantages in terms of PHA isolation and downstream processing. Disruption of the cells can be performed without the involvement of any solvent, surfactant, or sodium hypochlorite solution. The exposition of halophilic archaea to deionized water, normally growing at a high salt concentration, triggers the rupture of the cell membrane and the release of all cellular components including PHA granules [74,75], minimizing environmental pollution and contamination of the biopolymers and contributing to PHA purification cost reduction.

### 3.3. Characterization of the Composition and Distribution of Monomers

Two kinds of analysis are suitable to determine the chemical composition and distribution of monomers in PHA: chromatographic techniques and spectroscopic techniques. To conduct chromatographic analysis, the PHA must be previously depolymerized into its monomers and oligomers, while spectroscopy is a non-destructive method that can be performed on the intact polymer. The next paragraphs summarize the main features of the techniques used to date for this characterization.

^1^H and ^13^C NMR (nuclear magnetic resonance) spectra of PHA are performed in polymer solutions in deuterated chloroform CDCl3 (solvent containing no protons) [76]. The polymer sample can absorb electromagnetic radiation within the radio frequency (rf) under appropriate conditions in a magnetic field. The frequencies of the absorption peaks are functions of certain nuclei in the molecule [77]. Several nuclei are magnetically active with a spin number of 1/2 and a uniform spherical charge distribution, being by far the most widely used ^1^H and ^13^C. In ^1^H NMR, the peak area is proportional to the number of protons it represents, and the spectrum shows a series of absorption peaks representing protons in different chemical environments. As a result, every portion of the spectrum can be associated with a certain monomer. ^13^C NMR will help to identify the functional groups like the -C=O group [78].

Fourier transform infrared spectroscopy (FTIR) is used to identify organic functional groups by measuring the absorption of infrared radiation as a function of wavenumber. Infrared radiation is absorbed and converted by an organic molecule into energy of molecular rotation and molecular vibration. The absorption of hydroxyl groups (–OH) in PHAs is observed in the range of 3460 to 3407 cm^−1^, while carbonyl (–C=O) and unsaturated ester (–COO) groups are observed within the range of 1742–1709 cm^−1^ [15]. The intensity of the methylene band near 2925 cm^-1^ provides additional information for PHA characterization [79].

High-performance liquid chromatography (HPLC) and gas chromatography (GC) require previous acid or alkaline hydrolysis or ethanolysis of the polymer into organic acids or esters. Both flame ionization detection (FID) and mass spectrometric (MS) detection are used for GC analysis of PHA monomers and oligomers [76,79].

### 3.4. Analysis of Molecular Weight Distribution

The average molecular mass of the PHA polymers (M_w_), molecular mass distribution (M_n_), and polydispersity index (PDI; M_w_/M_n_) can be determined through a gel permeation chromatography (GPC) system [27,46], with a separation mechanism based on the size of the polymer molecules in solution rather than on the chemical properties. To conduct this kind of assay, the polymer sample is first dissolved in a solvent, forming coil conformation. These coiled up polymer molecules are then introduced into the mobile phase and flow into the GPC/SEC column. Through this structure, small polymer coils (that can enter many pores in the beads) take a long time to pass through the column, while large polymer coils that cannot enter the pores take less time to leave the column. To determine the molecular weights of the components of a polymer sample, a calibration with standard polymers of known weight must be performed to relate retention to molecular weight. GPC can be coupled to light scattering or viscosity analysis for further characterization [79].

### 3.5. Characterization of Thermal Properties

Thermogravimetric analysis (TGA) and differential scanning calorimetry (DSC) are thermal analysis techniques widely used to characterize polymers. In TGA, the sample is heated while measuring the weight, obtaining a time temperature curve versus sample weight and recording the loss of mass at different temperatures. DSC is a thermal analysis technique in which the heat flow, into or out of a sample, is measured as a function of temperature or time while the sample is exposed to a controlled temperature program (heating or cooling). Exothermic phenomena, such as decomposition or crystallization, can be studied by DSC as well as endothermic changes such as melting or glass transition [80]. DSC is used to determine melting temperature (T_m_), glass transition temperature (T_g_), degree and temperature of crystallization, and decomposition temperature (T_d_). Thermal stability of PHAs can be studied by TGA, recording the temperature at which the sample starts losing weight (degradation temperature), or by DSC, recording the time or temperature of degradation that corresponds to an exothermic heat flow [28].

### 3.6. Characterization of Crystallinity

The degree of crystallinity of PHAs highly influences their mechanical properties. Two analysis techniques can be used to study the crystallinity of polymers: DSC and X-ray powder diffraction (XRD) analysis. X-ray powder diffraction measurement helps to understand the crystalline nature and morphology of PHAs [78]. All crystalline materials have one thing in common: their components (atoms, ions, or molecules) are arranged in a regular manner. The incident X-ray beam is scattered at different planes of the material and reflected X-rays are detected; the result of the measurement is a so-called diffractogram. This is a plot of X-ray intensity on the y-axis versus the angle 2θ (2θ is defined as the angle between the incident and the diffracted beam) on the x-axis. Amorphous regions of the samples produce broad peaks, whereas crystalline regions produce sharp peaks [81]. The degree of crystallinity (X_c_) can be determined by determining the intensities of the crystalline (I_c_) and amorphous (I_a_) contents in the sample.

## 4. Organisms Producing Bioplastics and Their Use as Cell Factories for PHA Production

It has been extensively described in the literature that most PHAs/PHBs are synthesized and accumulated as carbon and energy storage materials in various groups of microorganisms, such as eubacteria, cyanobacteria, and archaea. Depending on the organisms and the growing conditions, different polymers are produced [82]. As examples, factors like pH and types of carbon source influence the molecular masses of PHAs [83,84,85].

**Figure 2:**
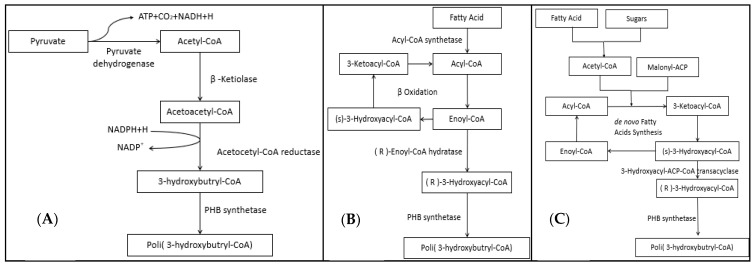
Main metabolic pathways for PHA synthesis. (**A**) synthesis from pyruvate; (**B**) synthesis through beta-oxidation of fatty acids; (**C**) synthesis through the pathway of fatty acid synthesis.

A deep revision of the literature reported over the last two decades focused on how PHA synthesis revealed that microorganisms able to synthesize these biopolymers may use various metabolic pathways depending on the nature of the initial substrate available in the culture medium (Figure 2). However, the initial metabolite in the three main pathways described from microbes is acetyl-CoA. When the main source in the microbial culture media is a carbohydrate, this compound is metabolized up to pyruvate, which is finally used for the synthesis of PHAs [86]. In this metabolic pathway, a β-ketotylolase catalyzes a reaction in which an acetyl-CoA binds to another acetyl-CoA or to a propionyl-CoA. In the following steps, NADPH is oxidized to support the activity of an acetoacetyl-CoA reductase, and then polymerization occurs because of a PHA synthase. Short-chain PHAs are obtained in this path [27]. If the main source in the microbial is of lipid nature, beta oxidation or fatty acid synthesis can be activated [86]. In both cases, acetyl-CoA is the starting molecule, and the final compound is the same that polymerizes PHA, differentiating only in that the synthesis pathway generates the intermediate metabolite (R)-3-hydroxycil-CoA with the enzyme acyl-ACP-CoA transacylase, while the beta oxidation uses the intermediate enoyl-CoA together with the enzyme enoyl-CoA hydrolase. PHA molecules synthetized in both cases are longer than those obtained from carbohydrates as main sources [27].

**Figure 3:**
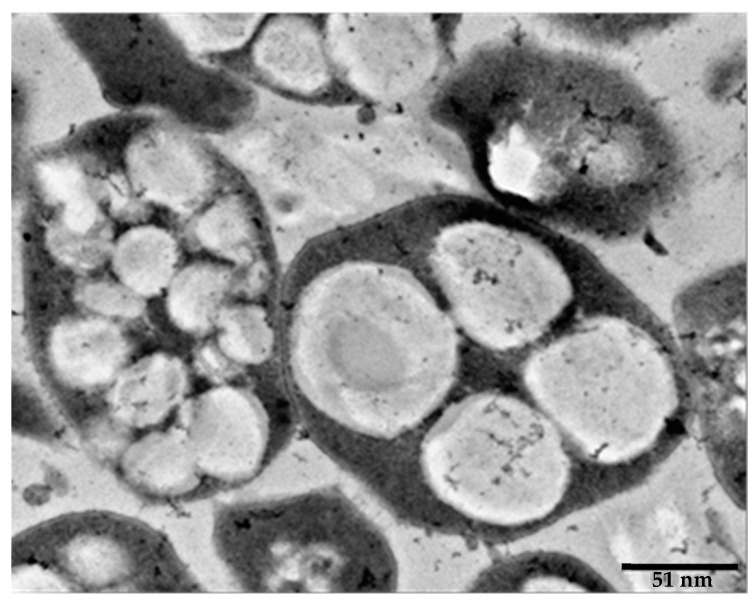
Electronic microscopy picture of *Haloferax mediterranei* cells showing PHA granules.

Accumulation of PHA/PHB polymers mainly occurs as intracellular inclusions (granules type) within bacteria as a result of their lipid nature as well as hydrophobicity. PHAs are agglomerate, reaching levels as high as 90% (w/w) of the dry cell mass [87] (Figure 3). The granules within the bacteria formed by surrounding the PHAs polymers with a monolayer of phospholipids combined with some proteins and with phasins being the most abundant molecules. This molecular strategy prevents the interaction of PHAs with water within bacterial cells, thus forming more stable crystalline polymers [88]. Consequently, phasins located on the surface of hydrophobic core in PHA granules contribute to the number and size of the PHA granules [89].

One important difficulty for broad manufacturing and commercialization of PHAs/PHBs is matching their production cost with that of petrochemical-based plastics. Lately, a considerable amount of effort has been dedicated to decreasing the production cost of PHAs/PHBs by using different approaches, such as raising effective bacterial strain and improving fermentation [90]. Advances in microbiology and molecular biology techniques have revealed the existence of bioplastic-producing species, which can be used as cell factories. Cells potentially used as cell factories successfully are mostly bacteria and, to a lesser extent, archaea. Bioplastics derived from transgenic plants are also gaining prompt significance for the large-scale production of PHAs/PHBs at low costs in terms of time and overall cost. Examples of organisms able to synthesize PHAs are mentioned in the subsections presented below.

### 4.1. Bacteria

Several bacterial strains were found to be associated with PHB accumulation among Gram positive [91], Gram negative bacteria [92], and photosynthetic bacteria [93], including cyanobacteria [94]. As example of PHA/PHB synthesizing bacteria, the following genera can be highlighted: *Pseudomonas, Bacillus, Citrobacter, Enterobacter, Klebsiella*, and *Escherichia* [95]. In addition, PHB production is widespread in nitrogen-fixing species, such as *Rhizobium leguminosarum*, *R. galegae*, *R. sp. hedysarum*, *R. meliloti*, *Azotobacter beijerinckii*, *A. macrocytogens*, and *A. vineandii* [96,97,98,99].

Depending on the culture conditions favoring PHA accumulation, bacteria are classified into two groups: (i) Bacteria requiring an excess carbon source and limiting essential nutrients such as oxygen and nitrogen for efficient PHA synthesis. The representative bacteria belonging to this group are *Cupriavidus necator*, *Protomonas extorquens,* and *P. oleovorans*. (ii) Bacteria that can accumulate PHA during the exponential phase and do not need nutrient limitation, such as *Alcaligenes latus*, *Azotobacter vinelandii,* and recombinant *E. coli* containing the PHA biosynthetic operon of *C. necator* [100]. The cultivation conditions for PHA biosynthesis are essential requirements for the development of cultivation techniques for large-scale production of PHA. Moreover, methylotrophs have been found to produce PHB but in a low yield [101].

In addition, PHA producers requiring moderate concentrations of salt in domain bacteria belong to the *Halomonadaceae* family, of which *Halomonas* is known to accumulate (scl)-PHA. Most *Halomonas* species have lower NaCl requirements (3–15%) for optimal growth. An alkaliphilic halophile, *Halomonas* sp. KM-1, was reported to produce PHB using glycerol as sole carbon source [102]. Moreover, by using CO_2_ and sunlight as both carbon and energy sources, cyanobacteria are known to accumulate PHA since they have the PHA synthase enzyme. In most cyanobacteria, only the PHB homopolymer has been identified to date.

### 4.2. Archaea

Within the Archaea domain, some genera belonging to the haloarchaea group have been the only ones described as PHA/PHB producers at the time of writing this review [103]. Haloarchaeal cultures at the moderate or large scale do not need strict sterile conditions due to the high concentrations of salt needed by the cells to be alive. This makes cultivation easier and more convenient compared to eubacterial strains. Besides, raw materials like brines could be even used to prepare culture media for these strains, thus contributing to circular economy processes [104].

Furthermore, cell walls and membranes of haloarchaea are easy to lyse in the absence of salt, particularly by using distilled water. This osmotic shock allows for the recovery of PHA, PHB, and PHBV granules from crude extracts in a much easier and more economical way [105,106]. In Section 5, detailed results regarding haloarchaeal species synthesizing bioplastics and their advantages are provided.

### 4.3. Eukarya

PHB synthesis from eukaryotic cells has not been described in detail at the time of writing this review, although it has been reported that yeast and some other eukaryotic microbial organisms contain small amounts of low-molecular-mass PHBs that act as polyphosphate complexes in membrane transport. In addition, the production of PHBs, especially in plants through genetic manipulation, is assessed as a potentially inexpensive alternative to prokaryotic production [107]. PHB production was mainly reported from *Saccharomyces cerevisiae*, *S. diastaticus, Candida krusei*, *C. tropicalis*, *Kloeckera apiculata*, *Kluyveromyces africans*, *K. lactis*, *Rhodotorula glutinis*, and *Ralstonia eutropha* among others [108,109].

On the topic of plants, recent studies demonstrated that some plants and invasive weeds are PHAs producers [110,111]. Moreover, it has also been confirmed that not only metabolites produced by plants but also plant biomass could be used as raw materials to produce PHB using eukaryotic microorganisms, thus supporting the development of biotechnological-based processes, which are promising and economically feasible [112]. However, most of these processes have only been explored at the laboratory scale. More work must be conducted in the near future to improve the knowledge in this specific field.

## 5. Advantages of Using Halophilic Microbes That Are Able to Produce PHA and PHB. Haloarchaea as Case of Study

Large-scale PHA/PHB production is an expensive process involving in most cases the use of organic solvents during the downstream processes (consequently, they are considered non-environmental biotechnological-based approaches). Mesophilic bacteria are vulnerable to contamination compared to extremophiles, and it is required to select robust production strains capable of growing in extreme conditions in which most of organisms could not proliferate. This is an important aspect to consider when designing large-scale biotechnological approaches. Consequently, extremophiles have been recently identified as the most promising microorganisms to produce PHA, reducing production costs [113]. Specifically, halophilic microbes show many advantages over non-halophilic microbes. Some of the main advantages are as follows: cell lysis is easy by exposing the cells to distilled water; many haloarchaeal species show fast growth compared to other microbial strains; sterilization is not essential because most common microbes used in lab (mesophiles) cannot grow under salty conditions; and, finally, waste materials like brines or salty waste waters from other processes could be used as culture media, thus minimizing the cost of the PHAs production.

Most halophilic archaea described to date can synthesize PHBV from structurally unrelated carbon sources, including starch, glucose, and glycerol [114]. Several studies show PHA production by haloarchaea genera, such as *Halococcus*, *Haloferax*, *Halorubrum*, *Halobacterium*, *Natronobacterium, Natronococcus, Halopiger*, or *Haloarcula* [66,115,116,117]. The genus *Haloferax* is of special interest due to its growth rate and high PHA productivity, the high quality of this PHA, and its substrate spectrum [118]. Specific information about haloarchaea as PHA producers is summarized in the following subsections.

### 5.1. Haloarchaeal Species Capable of Producing PHA, PHB and PHV

PHA accumulation in haloarchaeal cells was firstly described from *Halobacterium sp.* (currently known as *Haloarcula marismortui*) by Kirk and Ginzburg (1972) [119], half a century after the discovery of PHA by Lemoigne (1923) [20]. Since that time, more PHA-accumulating haloarchaea have been described. Fernandez-Castillo et al., in 1986 [120], found PHB in three species of the *Haloferax* genus and (*Hfx. mediterranei*, *Hfx. volcanii*, *Hfx. gibbonsii*) and in *Haloarcula hispanica*. Shortly after this work, accumulation of PHA was also observed in two more species of *Haloarcula* genus (*Har. vallismortis* and *Har. japonica*) [121,122]. *Halopiger aswanensi* was subsequently found to accumulate PHB for around 53% of its cell dry weight by using n-butyric acid and sodium acetate as sources of carbon [123,124]. Two species of *Haloquadratum walsbyi* as well as other species belonging to the following genera have been described as PHA producers: *Halostagnicola, Haloterrigena, Halobiforma, Haloarcula, Halobacterium, Halocococcus, Halorubrum, Natrinema* and haloalkaliphiles that include *Natronobacterium* and *Natronococcus* [66,125,126,127,128]. The third most abundant species in Antarctica’s Deep Lake, *Halorubrum lacusprofundi*, was recently confirmed to be a PHA-like granules-producer at low temperatures [129].

*Hfx. Mediterranei* is probably the most preferred PHA producer among all the haloarchaeal strains due to its high growth rate, metabolic versatility, genetic stability, and effective transformation system [130]. Many studies show that *Hfx. Mediterranei* can use many industrial and household wastes as carbon sources to synthesize PHA with significant productivity. In addition, PHA synthesized by *Hfx. Mediterranei* is a copolymer of 3-hydroxybutyrate (3HB) and 3-hydroxyvalerate (3HV) from structurally unrelated substrates [131]. PHBV is a more versatile and economically favorable polymer than PHB [132]. Many species need precursor (3HV) for the synthesis of PHBV, while *Hfx. Mediterranei* can synthesize PHBV efficiently without such a precursor; therefore, this is another advantage of reducing the cost of its production [133]. Moreover, *Hfx. Mediterranei* is also capable of synthesizing poly(3-hydroxybutyrate-co-3-hydroxyvalerate-co-4-hydroxybutyrate) (PHBV4HB) in the presence of 4HB precursors in the medium (Y-butyrolactone) [134]. In brief, *Hfx. Mediterranei* has many advantages as a cell factory for the large-scale production of PHBV. Other strains, such as *Har. hispanic, Hgm. borinquense*, and *Natrinema* species, have also been found to accumulate PHBV by using unrelated substrates of carbon [28,135,136,137]; for instance, by using glucose as the sole source of carbon, *Natrinema ajinwuensis* accumulated PHBV with 13.93 mol% 3HV [138]. Moreover, *Halogranum amylolyticum* could efficiently accumulate PHBV at 20.1 mol% 3HV [139]. However, these haloarchaeal species, unlike *Hfx. mediterranei*, have disadvantages as a platform for further optimization of PHA production, such as low precursor for PHBV (3HV) content, slow growth rate, excess salinity requirements, or the absence of a manageable genetic transformation system. Nevertheless, these studies suggest that more haloarchaeal species with PHBV synthesis capability are being found [140].

### 5.2. PHA-Related Genes in Haloarchaea

Most of the haloarchaeal genomes are neither fully sequenced nor fully annotated [141]. This constitutes a significant limitation when bioinformatics approaches are used to explore genes and sequences related to specific metabolic processes. However, some molecules studies have been conducted regarding PHA synthesis.

The key enzyme involved in biosynthesis of PHA is PHA synthase catalyzing the polymerization of the hydroxyalkanoate monomer to produce PHA chains. PHA synthase in haloarchaea consists of two subunits, PhaC and PhaE, and belongs to class III. PHA synthase in halophilic bacteria, in contrast, consists of only the PhaC subunit and belongs to class I [140]. In haloarchaea, PHA synthase possesses some novel features, as shown below.

The first archaeal PHA synthase activity was detected by Hezayen and co-workers et al. (2002) from the halophilic archaeon called strain 56 [126]. PHA synthase was associated covalently with PHB granules, and the expression of the genes coding for it was only induced under nutritional conditions promoting PHB accumulation. However, molecular characterization of the enzyme was not possible due to lack of the sequence similarity. Using complete genome sequence of *Har. marismortui*, Han and co-workers identified and characterized PHA synthase genes [142]. *Har. hispanica*, a haloarchaeon phylogenetically close to *Har. marismortui*, was also analyzed in this study, finding highly homologous *phaEC* genes present in the genome and PHB production up to 9.9% (wt) [142]. Only *phaC* and *phaE* co-expression in the Δ*phaEC* mutant strain restored PHA accumulation of *Har. hispanica*. However, it has been found that the PhaC protein is stably attached to the PHA granules, whereas PhaE is not. This observation suggests that in *Haloarcula* species, the PhaC and PhaE subunits constitute a novel type of class III PHA synthase.

Likewise, PHA synthase of *Hfx. mediterranei* also consists of two subunits of PhaE and PhaC, and both proteins are constitutively expressed in nutrient-limited and nutrient-rich media [135]. *phaE* and *phaC* genes were also reported in *Hgn. amylolyticum* by Zhao et al. (2015) [139], and the PhaE and PhaC amino acid sequence showed 64% and 62% identity with *Hfx. Mediterranei* proteins, respectively. PhaC has been detected in *Hrr. Lacusprofundi*; this haloarchaeon produces PHA at low temperatures [129]. The molecular weight of PhaE and PhaC from haloarchaea was found to be 20 kDa and 50.1 to 58.5 kDa respectively, differing from that of the bacterial class III PHA synthase (40 kDa) [106]. Haloarchaeal PhaC consists of a longer C-terminal as compared to bacterial PhaC [66,106,127,129,131,133,135]. This longer C-terminus is essential for activation of PHA synthase because its truncation results in less PHA accumulation.

Furthermore, unlike the bacterial PhaE subunit, PhaE-box was present only in *Haloarcula* species and *Halorhabdus utahensis* DSM-12940, suggesting that this box is not so preserved in PhaEs. The phylogenetic analysis of PHA synthase from haloarchaea and bacteria clearly clustered them into two distinct domains [106]. This strongly suggests that PHA synthase from the haloarchaea is a novel subtype of class III PHA synthase. In addition to the *phaC* gene clustered with *phaE*, three additional *phaC* paralogues (designated as *phaC1*, *phaC2,* and *phaC3*) were described in *Hfx. Mediterranei* [106]. Although during PHBV accumulation the three additional genes were not transcribed, expression of PhaE from *Hfx. mediterranei* led to accumulation of PHBV with a varied 3HV content. Phylogenetic analysis based on PhaCs *Hfx. mediterranei* and PhaCs from other haloarchaeal species indicated that three additional PhaCs may have evolved through horizontal transfer from other sources and not directly from *Hfx. Mediterranei* PhaCs [140].

### 5.3. Effects of Cultivation Conditions on the Production of PHAs

Accumulation of PHAs as intracellular carbon and energy storage granules is one of the main strategies used by organisms that are PHA producers. This is in fact a stress response employed by many microorganisms and plants to adapt to their environment. Thus, an excess of carbon substrate and a deficit of other elements, such as nitrogen and phosphorus, are the nutrient-limiting stress conditions that induce the synthesis of PHA [143]. The carbon source accounts for up to 50% of production costs, and for that reason, great efforts have been made to identify nutritional conditions based on inexpensive raw materials, such as industrial waste streams, to find low-cost and feasible culture media to upscale in terms of mid–large-scale production of these biopolymers. Table 3 summarizes the most important works in which different raw materials and waste materials from different industrial activities have been used to grow haloarchaeal species that are able to produce PHAs. Haloarchaea are very versatile microorganisms in terms of carbon sources for PHA production, and the waste streams utilized for that purpose include hydrolyzed whey from dairy industry, pretreated vinasse from the ethanol industry, olive mill wastewater, rice-based ethanol stillage, crude glycerol from the biodiesel industry, enzymatic extruded starch, date palm sugars, seaweed hydrolysate, and sugarcane bagasse, among others. Of note, haloarchaea from the genus *Haloarcula* sp. can accumulate up to 46.6% PHA in DCW using petrochemical wastewaters as a carbon source [144]. Most haloarchaea can synthesize the copolymer PHBV even without any external precursor, and they also contribute to reducing the production cost. The 3HV molar fraction in PHBV produced from glucose as the sole carbon source reached up to 9% in *Haloferax mediterranei* [132] and exceeded 20% in *Halogeometricum borinquense* E3 [28]. Moreover, the 3HV molar fraction can reach up to 99% in *Haloferax mediterranei* in the presence of direct precursors in the culture media, such as in the case of volatile fatty acids [145].

## 6. Global Market for Bioplastics

During the 80s and 90s of the last century, attempts were made to reduce the volume of waste in landfills, and, opening a market opportunity, the idea of developing biodegradable materials became a topic of interest. The first biodegradable plastics developed took too long to degrade and were not well received [156]. Currently, biodegradable bioplastics have reached great maturity. The new biodegradable polymers are susceptible to biodegradation by microorganisms, and they are developed for specific markets, such as agriculture, food packaging, and composting bags.

It should be noted that the compostable aspect refers to plastics that are capable of biodegrading in a certain period. From an industrial point of view, the compostable aspect is more important than the biodegradable aspect. The term compostable is defined by ASTM D6400-19 [157] and EN 13432 [158] standards in such a way that compostable is a concept measurable in time, while the biodegradable concept refers to a property without a clearly defined temporal durability. Labeling (certification) programs based on the ASTM Standard D 6400-19 (North América) or EN 13432 (Europe) have been developed for materials suitable for composting applications. In this sense, all compostable plastics are biodegradable, but not all biodegradable plastics are compostable. A compostable plastic is an unavoidable requirement in a market that the increasingly greener consumer has concerns about. For this reason, all bioplastic producers are trying to commercialize compostable and therefore biodegradable bioplastic.

Currently, biodegradable plastics can be obtained from renewable sources but also from fossil sources. Thus, PLA, PHA, and starch mixtures are of renewable origin, while polyesters, such as PBAT or PBS, come from non-renewable sources. Thermoplastic starch blends and PBAT-type polyesters are widely used in mulches for agricultural applications [159], where the collection and recycling of plastics is difficult, and the use of compostable polymers is more convenient. On the other hand, considering the increasing environmental awareness of the consumer, PLA [160], and to a lesser extent PHA [34], is mainly used in applications for food packaging with the aim of reducing the carbon footprint of this type of product.

The bacteria production of PHA was discovered in 1920. Nowadays, it is well known that a special polyester called poly(3-hydroxybutyrate) is accumulated like intracellular granules as carbon and energy reserves in a variety of Gram-positive and Gram-negative bacteria [161]. Today, polyhydroxyalkanoates are one of the most studied biodegradable and bio-based polymers with the greatest potential for development in the coming years. Not only does PHA play an important role in the packaging or agriculture, but it also great potential in the medical sector. Due to its high mechanical strength, it is extensively used for the preparation of medical scaffolds in the form of screws or pins [78].

**Figure 4:**
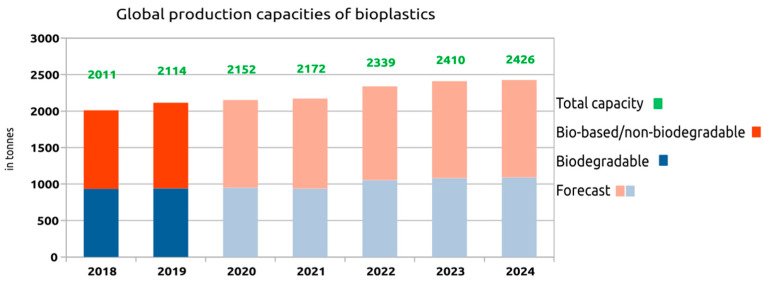
Current and forecast global production capacities of bioplastics [163].

To put into context the relative importance of PHA polymers in the plastic industry, it is necessary to consider that world plastics production in 2018 according to the Association of European Plastics Manufacturers (Plastics Europe) [162] was 359 million tons. Of this amount, 17% was in Europe, 18% in North America, and 51% in Asia. On the other hand, the global production of bioplastics according to the Association of European Bioplastic Manufacturers (European Bioplastics) [163] reached a total of 2.11 million tons in 2019. Of this amount, 0.94 million tons correspond to non-biodegradable bioplastics and 1.17 million tons to biodegradable plastic. Therefore, bioplastics represent a percentage of just 0.6% compared to traditional plastics. However, the forecast is that by 2024, the production of bioplastics will reach a total of 2.43 million tons with a smooth but sustained growth that indicates its increasing importance (Figure 4).

**Figure 5:**
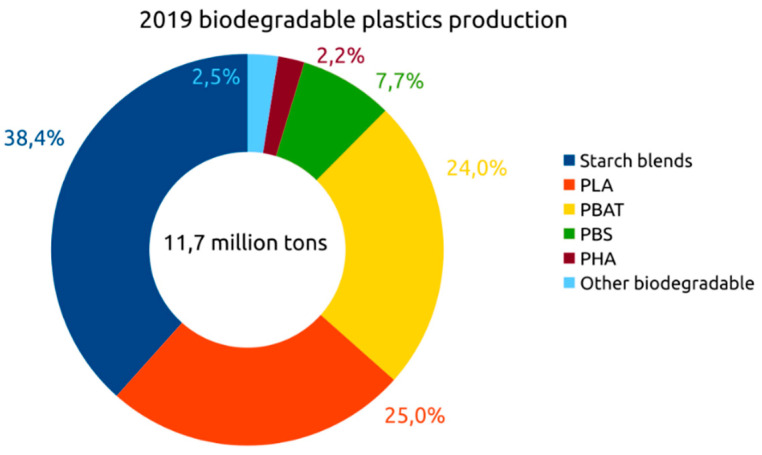
2019 biodegradable plastic production [2].

Looking at the production capacity and specifically the type of biodegradable bioplastic (Figure 5), it is possible to conclude that of the total of 11.7 million tons, starch mixtures dominate the market with 38%, followed by PLA and PBAT with approximately 25% each, and PHA boasts a modest 2%. However, according to the European Association of Bioplastic Manufacturers [163], PHA is one of the most important biopolymers, whose production capacity will triple in the next 5 years.

**Figure 6:**
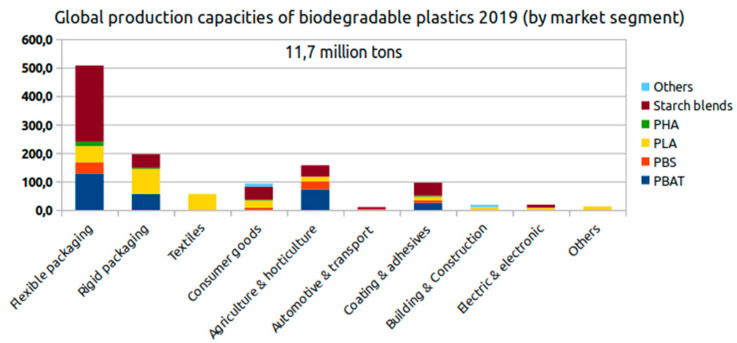
Global production capacities of biodegradable plastics 2019 (by market segment).

The market for biodegradable plastics is constantly growing, and new applications appear every day. In this sense, it is possible to identify the most important markets for biodegradable plastics where both rigid and flexible packaging containers are the most important field of application (Figure 6) [163].

Although there is a lot of research being conducted regarding PHAs and their derivatives at different laboratory and pilot plant stages, there are not many producers of these biodegradable biopolymers at the industrial stage. Table 4 shows the current PHA producers with PHA in the market.

## 7. Conclusions

Microbiologically produced PHAs are viable candidates to replace conventional oil-based plastics. However, the production costs of this biopolymer are still considerably higher in comparison to those of traditional polymers. Important challenges should be overcome, including the costs of the carbon source as well as enhancing the production and extraction efficiency. In these terms, haloarchaea species provide the advantage of utilizing inexpensive carbon sources of industrial origin, lower energy requirements due to negligible sterility precautions, downstream processing without the use of any chemical solvent for cell lysis, the recyclability of process side-streams (spent fermentation broth and cell debris), and the production of 3HV-containing copolyesters from unrelated carbon sources. Promising findings regarding haloarchaea-related PHA production have been reported in recent years at the laboratory scale. However, very few works have provided an in-depth characterization at the pilot or semi-industrial scale, and marketable prototypes from haloarchaeal PHA and techno-economic assessments are scarce. What is needed now is to drive the upscaling of those promising processes at the lab-scale to boost the development of archaeal cell factories and illustrate their potential in the future of sustainable biotechnology.

## Figures and Tables

**Table 1:** Monomers that can be found in the molecular structure of PHAs.

Monomer	Abbreviation	Group (R)	N° of Carbons	Chemical Structure
3-hydroxypropionic acid	3HP	Hydrogen	3	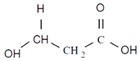
3-hydroxybutyric acid	3HB	Methyl	4	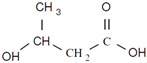
3-hydroxyvaleric acid	3HV	Ethyl	5	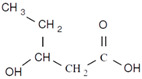
3-hydroxyhexanoic acid	3HHx	Propyl	6	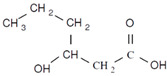
4-hydroxybutiric acid	4HB	Hydrogen	4	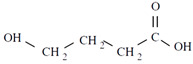
4-hydroxyvaleric acid	4HV	Methyl	5	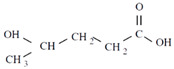

**Table 2:** Summary of the properties of some of the most marketed bioplastics [34,35].

Name	Chemical Structure	Physicochemical Properties
Polyhydroxyalkanoate (PHA)	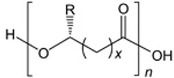	Water-insoluble; UV resistance; poor resistance to acids and bases; soluble in chloroform and other chlorinated hydrocarbons.
Polyhydroxybutyrate (PHB)	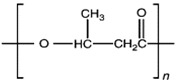	High crystallinity degree; brittle.
Polyhydroxyvalerate (PHV)	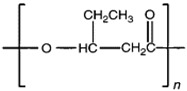	PHB co-polymer; lower crystallinity degree and more flexibility than PHB.
Poly(3-hydroxybutyrate-co-3-hydroxyvalerate) (PHBV)	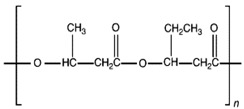	Brittle; high elastic modulus; low tensile strength.

**Table 3:** Summary of the main studies using haloarchaeal species as cell factories to produce PHAs. Analytical grade culture formulations or industrial and agricultural residues have been used as substrates for the preparation of culture media ensuring both optimal cell growth and the production of PHAs.

Strain	Carbon Source	Type of PHA (mol %)	Cultivation Mode	DCW (g L^−1^)	PHA (g L^−1^)	PHA/CDW (%)	Y_PHA/Subst_	Productivity (g L^−1^ h^−1^)	Ref.
*Haloferax mediterranei DSM 1411*	25% pretreated vinasse	PHBV (12.4%3HV)	Flask		19.7	70.0	0.87	0.21	[146]
*Haloferax mediterranei DSM 1411*	50% pretreated vinasse	PHBV (14.1%3HV)	Flask		17.4	66.0	0.52	0.18	[146]
*Haloferax mediterranei DSM 1411*	Hydrolyzed whey	PHBV (6.0% 3HV)	Batch-42L Bioreactor		12.2	72.8	0.29	0.09	[134]
*Haloferax mediterranei DSM 1411*	Hydrolyzed whey + sodium valerate + Y-butyrolactone	P-(3HB-co-21.8%3Hvco-5.1%4HB	Batch-10L Bioreactor		14.7	87.5	0.20	0.14	[134]
*Haloferax mediterranei DSM 1411*	MST medium with 15% of olive mill wastewater	PHBV (6.5% 3HV)	Flask	10	0.2	43.0	n.r.	n.r	[147]
*Haloferax mediterranei DSM 1411*	Rice-based ethanol stillage	PHBV (17.91%3HV)	14 L tank		13.2	63.0	0.27	0.14	[148]
*Haloferax mediterranei DSM 1411*	Crude glycerol (biodiesel industry)	PHBV (10% 3HV)	Fed batch10L Bioreactor		13.4	75.4	0.37	0.12	[115]
*Haloferax mediterranei DSM 1411*	Hydrolyzed cheese whey	PHBV (98.5%HB–1.5%3HV)	Batch bioreactor	7.6	7.92	53.0	0.78	0.17	[75]
*Haloferax mediterranei DSM 1411*	Glucose + galactose	PHBV	Flask	6.8	6.7	46.0	0.66	0.055	[75]
*Haloferax mediterranei DSM 1411*	Enzymatic extruded starch	PHBV (10.4%3HV)	6L Fed-batch Bioreactor	39.4	20	50.8	n.r.	n.r.	[149]
*Haloferax mediterranei DSM 1411*	Butanoic: pentanoic VFAs (29:71)	PHBV (71.5%3HV)	Fed batch flask	5.8	1.5	25.0	0.14	n.r.	[145]
*Haloferax mediterranei DSM 1411*	Butanoic: pentanoic VFAs (56:44)	PHBV (44.4%3HV)	Fed batch flask	6.0	1.2	19.9	0.11	n.r.	[145]
*Haloferax mediterranei DSM 1411*	Butanoic: pentanoic VFAs (79:21)	PHBV (20.6%3HV)	Fed batch flask	5.5	1.2	20.7	0.11	n.r.	[145]
*Haloferax mediterranei DSM 1411*	0.5M pentanoic VFA	PHBV (99.5%3HV)	Fed batch flask	5.5	1.5	27.1	n.r.	n.r.	[145]
*Haloferax mediterranei DSM 1411*	0.5M propanoic VFA	PHBV (66.2%3HV)	Batch flask	6.7	1.0	14.5	n.r.	n.r.	[145]
*Haloferax mediterranei ES1* (engineered strain)	10 g/L glucose	PHBV (8.9%3HV)	Shake flask	10.3	3.3	32.4	n.r.	n.r.	[132]
*Haloferax mediterranei ES1* (engineered strain)	10 g/L glucose + 6.5 mM valerate	PHBV (20.8%3HV)	Shake flask	11.6	4.0	34.4	n.r.	n.r.	[132]
*Haloferax mediterranei ES1* (engineered strain)	10 g/L glucose + 15 mM valerate	PHBV (36.6%3HV)	Shake flask	13.3	5.4	41.0	n.r.	n.r.	[132]
*Haloferax mediterranei DSM 1411*	Ricotta hydrolyzed cheese whey	PHBV	Batch 3 L bioreactor	18.3	1.27	n.r.	0.1	n.r.	[150]
*Haloferax mediterranei DSM 1411*	Date palm sugars	PHBV (18%3HV)	2 L Fed-batch bioreactor	18	4.5	25.0	n.r	n.r	[151]
*Haloferax mediterranei DSM 1411*	Hydrolyzed whey permeate	PHBV (10%3HV)	220 L bioreactor	n.r.	7.2	66.0	n.r	n.r	[152]
*Haloferax mediterranei DSM1411*	25% v/v of macroalgal hydrolyzate (*Ulva sp*.)	PHBV (8%3HV)	Shake flask	3.8	2.2		0.55	0.035	[153]
Halogeometricum borinquense strain TN9	20 g/L glucose	PHB	Shake flask			14.0			[29]
*Natrinema ajinwuensis* RM-G10	Glucose	PHBV(13.9%3HV)	Batch flask			61.7		0.211	[138]
*Halogeometricum borinquense* E3	20 g/L glucose	PHBV(21.5%3HV)	Batch flask	2.1	n.r.	75.2	n.r.	0.025	[28]
*Haloarcula marismortui* MTCC1596	100% pretreated vinasse (ethanol industry)	PHB	Shake flask	15	4.5	30.3	0.77	0.021	[154]
*Haloarcula sp. IRU1*	Glucose	PHB	Shake flask			66.0			[155]
*Halogeometricum borinquense* E3	Sugarcane bagasse 25%	PHBV (13.3%3HV)	n.r.	n.r.	n.r.	50.0	0.44	0.0095	[136]
*Natrinema palladium 1TK1*	2% whey nutrient broth	PHBV	Shake Flask	0.41	0.20	47.7	n.r.	n.r.	[137]
*Natrinema palladium 1TK1*	2% tomato nutrient broth	PHBV	Shake Flask	2.8	0.87	31.2	n.r.	n.r.	[137]
*Natrinema palladium* 2KYS1	2% melon nutrient broth	PHBV	Shake flask	0.55	0.15	26.3	n.r.	n.r.	[137]
*Natrinema palladium* 5TL6	2% corn starch nutrient Broth	PHBV	Shake flask	0.43	0.18	41.4	n.r.	n.r.	[137]
*Haloarcula sp*	Petrochemical wastewater	PHB	Shake flask	n.r.	n.r.	46.6	n.r.	n.r.	[144]

n.r.: not reported; VFAs: volatile fatty acids.

**Table 4:** PHA industrially available.

Manufacturer	PHA Type	Reference
Kaneka Belgium NV	PHBH	[164]
TianAn Biopolymer	PHBV	[165]
Bio-on S.p.A.	PHA	[166]
Telles (a joint venture of Metabolix and ADM)	PHBV	[167]
Tianjin GreenBio Materials Co.	PHA	[168]
Danimer Scientific	PHA	[169]
Bluepha	PHA	[170]
CJ CheilJedang Corp.	PHA	[171]
Full Cycle	PHA	[172]
PolyFerm Canada	PHA	[173]
Mango Materials	PHA	[174]
Biomer	PHB	[175]
Newlight Technologies	PHA	[176]
